# Actinomyces Acute Rhinosinusitis Complicated by Subperiosteal Abscess in an Immunocompromised 12-Year-Old: Case Report and Literature Review

**DOI:** 10.1155/2022/7058653

**Published:** 2022-04-11

**Authors:** Sai V. Nimmagadda, Li-Xing Man, Margo K. McKenna, John J. Faria, Isaac L. Schmale

**Affiliations:** ^1^University of Rochester School of Medicine and Dentistry, University of Rochester Medical Center, Rochester, NY, USA; ^2^Department of Otolaryngology Head and Neck Surgery, University of Rochester Medical Center, Rochester, NY, USA

## Abstract

**Objective:**

To describe a rare case of pediatric actinomycotic rhinosinusitis with orbital subperiosteal abscess and review the current literature to assess methods of diagnosis, treatment modalities, and outcomes with appropriate treatment.

**Methods:**

A case report and a review of the literature.

**Results:**

A 12-year-old patient with Crohn's disease on infliximab presented with rhinosinusitis with orbital subperiosteal abscess formation. Endoscopic sinus surgery was performed and cultures grew actinomyces. A prolonged course of antibiotics was started, resulting in the complete resolution of the infection. In a literature review, all cases of uncomplicated and complicated actinomyces rhinosinusitis managed with appropriate surgery and prolonged antibiotics resulted in a cure. Our case is the first reported in a pediatric patient and the first taking immunosuppressive medication. Overall, only 3 cases of actinomyces rhinosinusitis in immunosuppressed individuals have been reported, each with uncontrolled diabetes and each also responded well to surgery and appropriate antibiotics.

**Conclusion:**

Actinomycosis of the paranasal sinuses poses a diagnostic challenge, with infections varying widely in presentation and extent of disease. A high index of suspicion, appropriate testing, and early aggressive treatment are critical in managing patients with this infection. Our case and prior published studies show that actinomyces rhinosinusitis can be successfully managed with endoscopic sinus surgery, abscess drainage as necessary, and a prolonged course of antibiotics, even in immunocompromised and pediatric populations.

## 1. Introduction

Common commensal organism in the oropharynx, actinomyces is a gram-positive anaerobe with pathogenic potential in the settings of immune impairment or mucosal injury [1]. With a reported incidence of fewer than 100 cases per year, it is a rare infection often complicated by delayed diagnosis and treatment onset due to low clinical suspicion [2]. *Actinomyces israelii*, the most common infectious actinomycotic species, usually presents in three forms: cervicofacial, pulmonary/thoracic, and abdominopelvic. Few cases reporting actinomycosis of the paranasal sinuses have been published, limited primarily to isolated case reports and small case series. Even less has been reported on complications of actinomyces infection of the sinuses in immunocompromised patients or within pediatric populations [3–5]. The occurrence of actinomycosis in the female pediatric age group as seen in our case is a rare entity as the disease generally has a peak incidence in the 4th to 6th decade of life with a slight male predominance and has no predilection for age, season, race, or occupation [6]. When found in children, it rarely spreads beyond superficial cervicofacial lesions and usually originates from a dental procedure or trauma. Immunosuppression can be a complicating factor, and while actinomycosis is not strictly considered an opportunistic infection, it has been described in individuals with impaired immune systems, as in HIV disease or leukemia, as well as diabetes and the use of immunosuppressive drugs [6, 7].

In this report, we describe an atypical case of progressive paranasal actinomycosis leading to the formation of a subperiosteal abscess in an immunocompromised child. We also performed an updated literature review to assess the variety of presentations, diagnostic approaches, treatment strategies utilized, and clinical outcomes. To our knowledge, paranasal actinomycosis with associated orbital complications have not been described in this patient population, and the following diagnostic and treatment strategy may help guide the management of future cases.

## 2. Case Presentation

**Figure 1:**
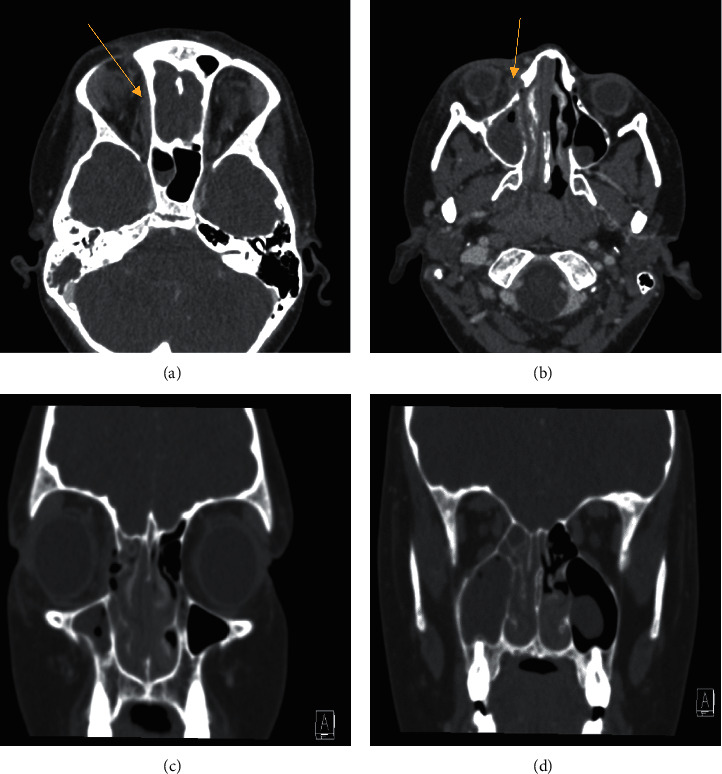
Computed tomography with contrast images of the presented case: (a) axial cut demonstrating a superior and medial subperiosteal abscess (yellow arrow). (b) a smaller rim-enhancing fluid collection inferior to the abscess in A. (c) & (d) coronal sections through the paranasal sinuses with notable mucosal thickening and mucosal thickening with air-fluid levels within the right maxillary sinus, right ethmoid air cells, and right sphenoid sinus.

A 12-year-old female with Crohn's disease on the antitumor necrosis factor monoclonal antibody infliximab presented with two weeks of upper respiratory symptoms. After one week, symptoms including purulent rhinorrhea, facial pressure, and nasal congestion worsened. After multiple febrile episodes, she was started on cefdinir by her pediatrician. Two days later, she presented to the emergency department for evaluation due to worsening eye swelling. On exam, she had edematous inferior turbinates with purulent drainage. The oral cavity examination was unremarkable without lesions, overt fistulous tracts, or identifiable dental caries. She acknowledged mild tooth pain but denied any history of recent dental procedures. Extraocular movements were fully intact bilaterally, but there was some right orbital pain as well as right upper and lower lid edema and erythema. Cultures from purulent nasal drainage were taken and subsequent computed tomography (CT) of the orbit, sella, and fossa with contrast demonstrated right maxillary and ethmoid opacification and associated orbital cellulitis with subperiosteal abscess formation (Figure 1). There were no obvious periapical lucencies. She was started on intravenous ampicillin/sulbactam and vancomycin and was admitted for further management. Exam the next day remained unchanged with orbital edema, erythema, and pain with extraocular movements. Given her immunocompromised status on infliximab, the patient was consented to endoscopic sinus surgery and drainage of the sub-periosteal abscess. The patient underwent anterior ethmoidectomy and maxillary antrostomy. Intraoperatively, a small defect of the lamina papyracea was identified but no obvious dehiscence or orbital fat or contents were appreciated. All purulence was drained and aerobic, anaerobic, and fungal cultures from the maxillary sinus were collected. Intraoperatively, it was difficult to appreciate anatomic relationships due to the degree of inflammation and infection, and the maxillary sinus floor mucosa was too edematous to discern potential odontogenic origin. The culture returned actinomyces species, and infectious disease (ID) was consulted. Vancomycin was discontinued per ID's recommendations due to lack of methicillin-resistant *Staphylococcus aureus* growth on culture and the patient continued on ampicillin/sulbactam until postoperative day five, when she was transitioned to high-dose oral amoxicillin/clavulanate following significant clinical improvement in periorbital edema and ecchymosis and normalization of eye exam per ophthalmology. Given sensitivity and culture data, ID recommended high dose amoxicillin/clavulanate for 24 hours prior to discharge home with a plan for a three-week course of amoxicillin/clavulanate 600-42.9 mg/5 ml suspension 16.7 mL twice daily, followed by three months of amoxicillin 400 mg/5 ml suspension 25 mL twice daily. Amoxicillin alone was chosen as actinomyces is known to be greatly responsive to beta-lactam antibiotics. Three months was chosen as the duration due to the slow growth of the actinomyces species on culture, with plans for reevaluation including repeat erythrocyte sedimentation rate and clinical examination. She tolerated the oral antibiotic regimen well. The postoperative course was notable for the development of right-sided dacryocystitis that was managed with topical erythromycin and warm compresses with complete resolution. At the two-month postdischarge follow-up, the patient had recovered fully without complication.

## 3. Materials and Methods

Details of the case were obtained from a chart review, and a literature review was conducted using two separate databases: PubMed and Web of Science. The following keywords were used in the updated search: “paranasal” or “sinonasal,” “actinomycosis,” “actinomyces,” “pediatric actinomycosis,” “invasive actinomycosis.” The search was not capped by a range of dates and the updated search took place in August 2021. Only full English articles and only those qualifying as cases of sinonasal actinomycosis were included in our study. Cases that did not meet the criteria upon title and abstract review by two authors (SVN and ILS) were excluded. Abstracts of other relevant articles identified upon review of citations of the included articles were also evaluated for inclusion.

## 4. Discussion

Actinomycosis is a rare cause of rhinosinusitis. Sinonasal actinomycosis specifically, a rare subset of cervicofacial actinomycosis, usually affects females in their fifth decade of life [2]. The pathophysiology of the disease is thought to involve an initial source of mucosal injury such as infection and trauma, which allows for bacterial invasion and access to subcutaneous tissues. An optimal environment after subcutaneous migration is provided by copathogens, typically reducing local oxygen tension and inhibiting host defense, thus promoting ideal conditions for actinomycosis growth and eventual dissemination through direct extension. Classically, actinomycosis forms abscesses with sinus tracts or fistulas extruding the well-described yellow sulfur granules. As actinomycosis of the paranasal sinuses is very rare, with only occasional descriptions in case reports, clinical characteristics have not been well established. Some often-noted presenting features include congestion, rhinorrhea, and sinus pressure, which mimic the features of chronic sinusitis. In a literature review of 20 cases of sinonasal actinomycosis, 35% of cases had experienced prior dental procedures in the form of implants or extractions. Objective findings noted on CT scan in these patients very commonly identify either unilateral or bilateral opacification of the maxillary sinus with focal calcified densities [2]. In a case series published by Woo et al., all cases of paranasal sinus actinomycosis underwent endoscopic sinus surgery with removal of purulence and abnormal/necrotic mucosa. The average length of follow-up was 33.7 months without recurrence in any of the patients [1].

Features such as responsiveness to antibiotics, presence of unique risk factors, and imaging findings can raise suspicion for an actinomycotic process, but a definitive diagnosis of actinomycosis requires confirmed presence of the pathogen on the histopathological exam and/or culture [8]. Prior studies indicate that risk factors for developing actinomycosis of the sinuses include prior dental procedures, oroantral defects, and immune deficiency [9, 10]. Classic imaging findings of paranasal actinomycosis generally involve opacification of the maxillary sinus, either unilaterally or bilaterally. In a case series of paranasal actinomycosis highlighted by Woo et al. and also shown in Table 1, all patients had unilateral total or subtotal opacification of the maxillary sinus with focal calcified densities [1]. However, actinomycotic infections can be mistaken for malignant processes on imaging due to the frequent appearance of an expansile mass, often with associated bony erosion [11]. Several retrospective studies have found that the final diagnosis of paranasal actinomycosis was ultimately made based on the visualization of sulfur granules in both the purulent and necrotic material found in the paranasal sinus and in the multiple biopsy sections of sinus mucosa [1]. Culture data is also diagnostic and recovery of Actinomyces species from an appropriately cultured or biopsied specimen, with incubation under strict anaerobic or at least microaerophilic conditions for a minimum of 14 days, is recommended [12]. In our case described above, the diagnosis was confirmed via intraoperative cultures with preoperative imaging of bony erosion and abscess formation as well as a lack of response to antibiotics, raising suspicion for an atypical pathogenic etiology.

Management of actinomycosis often varies considerably by institution, as there are no standardized protocols. However, endoscopic sinus surgery followed by long-term intravenous antibiotic therapy is the gold standard for the treatment of sinonasal actinomycosis [8]. Initial antibiotic therapy generally consists of high doses of intravenous penicillin G followed by long-term oral antibiotics [13]. Paranasal actinomycosis can rarely be managed with medication alone, and surgical approaches are generally required for progressing infections, as seen in our case with worsening orbital erythema and edema despite intravenous antibiotic treatment. Based on the small number of cases in the literature, actinomycosis of the paranasal sinuses complicated by abscess or bony invasion is managed with surgical debridement and abscess drainage as necessary [1, 14]. Actinomyces tends to invade only locally, and the avascular fibrotic wall of the lesion makes treatment with only antibiotics difficult [1]. Therefore, the surgical removal of the involved tissues is important for the successful treatment of paranasal sinus actinomycosis [1]. Postoperative intravenous antibiotics followed by long term oral antibiotic therapy with amoxicillin, ampicillin, or penicillin V is thought to reduce disease recurrence [15]. As shown in Table 1 and 2, there were no reports of recurrence among cases of paranasal actinomycosis managed with proper surgical debridement and antibiotic usage.

Treatment of paranasal actinomycosis in the setting of immunosuppression has not been thoroughly examined to date. In fact, the results of the updated literature review failed to identify any patients currently taking immunosuppressive medications, as was the case with our reported patient. Some were present with uncontrolled diabetes, which is known to inhibit the natural immune response against pathogens [16]. One specific case highlighted by Sakuma et al. identifies a case of nasal actinomycosis in an immunocompromised patient resulting in death from progression. The significance of this case is limited by the patient's noncompliance and refusal of potentially lifesaving surgical resection, but the patient's uncontrolled diabetes made infection control more challenging [8]. It raises the question of whether immunocompromised/fully immunosuppressed patients could benefit from a more aggressive approach to infection control with early operative management vs. prolonged medical therapy with varied antibiotic regimens.

The case presented in this report highlights that in immunocompromised hosts, rhinosinusitis from unusual organisms should be considered and prompt aggressive therapy instituted if conservative treatment fails or with concern for impending complications. When treated with appropriate surgery and antibiotic therapy, cases of actinomycotic rhinosinusitis in immunocompromised and pediatric populations can achieve long-term resolution/cure.

## 5. Conclusions

Actinomycosis of the paranasal sinuses poses a diagnostic challenge, with infections varying widely in presentation and extent of disease. A high index of suspicion, appropriate testing, and early aggressive treatment are critical in managing patients with this infection. Our case and prior published studies show that actinomyces rhinosinusitis can be successfully managed with endoscopic sinus surgery, abscess drainage as necessary, and a prolonged course of antibiotics, even in immunocompromised and pediatric populations.

## Figures and Tables

**Table 1:** Cases of paranasal sinus actinomycosis with complications.

Reference	Age/sex	Presentation	Medical history	Immune status	Treatment	Outcome
[4]	42/F	Right temporal headache (3 wks), negative for dental infection. Orbital CT-ill-defined mass isodense with the brain, in the posterolateral part of the right orbit, right afferent pupillary defect, central scotoma, right proptosis, ↓ right corneal sensation, and loss of right ocular ductions. Presumed orbital infection vs. pseudotumor.	Low visual acuity → Count fingers (CF)	Not reported	Failed treatment for presumed orbital infection vs. pseudotumor: dexamethasone, empiric antibiotics, high-dose oral steroid treatment Orbital exploration via lateral canthotomy approach, green, purulent drainage Metronidazole, penicillin V + probenecid	3-month follow-up: moderate right ptosis, mild right abduction weakness
[5]	62/F	Left upper eyelid swelling, forehead and unilateral headache, bilateral frontal sinus tenderness, and discharging fistula in the lateral aspect of the upper eyelid. CT-the destruction of both inner and outer table of the skull with sequestrum formation in the left frontal sinus	Diabetes, left oroantral fistula after tooth extraction 1 year ago.	Immunocompromised	Left Caldwell-Luc for maxillary sinus drainage and fistula repair. External approach to frontoethmoidectomy Oral amoxicillin/clavulanate (8 wks) Benzathine penicillin (6 mo.)	Resolution
[8]	54/M	Left purulent nasal discharge (1 month), swelling and sharp pain at the root of nose	Uncontrolled DM, chronic Hep C	Immunocompromised	IV ampicillin/sulbactam (6 days) Oral amoxicillin (lost to f/u) Surgical debridement of hard palate + nasal cavity IV panipenem/betamipron + vancomycin → IV penicillin G (10 days) → d/c due to side effects (lost to f/u)	Death from multiorgan failure and DIC from disseminated infection
[10]	37/M	Fever, severe frontal headache, left retrobulbar and hemifacial pain, nasal obstruction, diplopia, and absence of left lateral gaze	Unremarkable	Normal	Bilateral transethmoidal sphenoidectomy + extensive removal of the anterior left sphenoid wall. IV vancomycin (2 wks), ceftriaxone, levofloxacin, pantoprazole, methylprednisolone Oral amoxicillin/clavulanic acid (8 wks) + saline nasal irrigations	Resolution
[17]	18/M	Fever, frontal headache, and bilateral papilledema. CT-ring-enhancing cystic mass in the right frontal lobe	Recurrent rhinopharyngitis	Normal	IV ciprofloxacin-metronidazole Oral erythromycin x	Resolution
[18]	35/M	Left eye proptosis and ptosis. CT-left pansinusitis and subdural empyema, cerebral edema with midline shift	Dental extraction	Not reported	IV cefotaxime-metronidazole Bedside percutaneous twist-drill aspiration (25 cc purulent material cultured) Frontotemporoparietal craniotomy and superior orbitotomy Penicillin (2 wks.) Oral tetracycline (4 wks.)	Resolution
[19]	43/M	Episodic left facial and temporal pain (2 months), acute onset diplopia, abducens nerve palsy, CT, and MRI-bilateral cavernous sinus swelling	Dental caries	Not reported	Oral corticosteroids (for presumed Tolosa Hunt syndrome) MRI → return of cavernous sinus swelling → pterional craniotomy + V penicillin (8 wks) + oral penicillin (3 mo.)	Resolution

CT: computed tomography, ESS: endoscopic sinus surgery, and IV: intravenous, and MRI: magnetic resonance imaging.

**Table 2:** Cases of paranasal sinus actinomycosis-uncomplicated.

Reference	Age/Sex	Presentation	Medical History	Immune Status	Treatment	Outcome
[1]	51/F	Facial pain, headache, CT-haziness in the left maxillary, and ethmoid sinuses with calcific density apparent in maxillary sinus	Unremarkable	Normal	ESS: maxillary antrostomy, ethmoidectomy, and debridement Cephalosporin (1 mo) and roxithromycin (3 mo)	Resolution
[1]	47/F	Postnasal drip, nasal congestion and CT shows haziness in the left maxillary sinus. Calcific density apparent in maxillary sinus CT	Unremarkable	Normal	See Table 2 Row 1	Resolution
[1]	52/F	Postnasal drip, headache, sneezing, CT shows haziness in left maxillary and ethmoid sinuses. Calcific density apparent in maxillary sinus CT	Unremarkable	Normal	See Table 2 Row 1	Resolution
[1]	58/M	Bloody discharge, CT shows haziness in the left maxillary sinus. Calcific density apparent in maxillary sinus CT	Unremarkable	Normal	See Table 2 Row 1	Resolution
[1]	49/F	Cough, snoring, rhinolalia, CT shows haziness in the right maxillary sinus. Calcific density apparent in maxillary sinus CT	Unremarkable	Normal	See Table 2 Row 1	Resolution
[1]	50/F	Nasal congestion, hyperrhinorrhea, postnasal drip, and CT shows haziness in the left maxillary sinus. Calcific density apparent in maxillary sinus CT	Oroantral fistula secondary to facial trauma	Normal	See Table 2 Row 1	Resolution
[3]	41/F	Severe aching left-sided headache, R-sided lateral rectus weakness. Skull x-ray: eroded clival margins and cloudiness of the sphenoid sinuses. Arteriogram: increased blushing in the clival-basi sphenoid area + cavernous sinus thrombosis.	Electroshock therapy, depression, and pneumonia (2x)	Normal	Transseptal sphenoidotomy Transethmoidal + antral sphenoidotomy IV penicillin → phenoxy methyl penicillin (3 mo.) Isoniazid + tetracycline (4 mo.)	Clival cortex restoration, sinus aeration, and sphenoid sclerosis on CT at 3 mo. follow-up.
[9]	50/F	Right facial pain and tenderness, maxilla numbness, ear fullness, pressure, hearing loss. CT showed opacification of right maxillary sinus.	Dental extraction	Not reported	Right middle turbinectomy, maxillary antrostomy, anterior and posterior ethmoidectomy. Endoscopy + debridement Penicillin VK (7 wks) Doxycycline	Resolution
[14]	42/F	Left maxillary tenderness, mucosal inflammation in the left middle meatus	Osseointegrated dental implants, oroantral fistula, and refractory sinusitis	Normal	Left Caldwell-Luc for maxillary sinus debridement Endoscopic ethmoidectomy IV penicillin (3 wks), Oral penicillin (3 mo.)	Resolution
[20]	67/F	Facial swelling, pain	Refractory sinusitis	Normal	Penicillin (6 mo.)	Resolution
[21]	32/F	Nasal congestion, headache. CT-intrasinus hyperattenuating tissue in the sphenoid sinus, suggesting chronic fungal sinusitis	Otorrhea R radical mastoidectomy + empirical systemic + otic antibiotics	Not reported	Endoscopic exploration and drainage of the sphenoid sinus.	Not reported
[22]	25/F	Left cheek discomfort (8 months), plain film, and CT showed a metallic foreign body in the left maxillary sinus	Chronic allergic rhinosinusitis, dental prosthesis extraction 3yrs prior	Not reported	Left Caldwell-Luc → maxillary sinus irrigation with an aqueous solution of 1% gentian violet and a specimen sent to pathology Oral amoxicillin/clavulanate + deflazacort (2 wks) Amoxicillin/clavulanate (3 mo)	Resolution
[23]	47/F	Mild chronic pain in the left buccal region with a tender lesion and slight swelling. CT-left maxillary and partial ethmoid opacification and calcified fragment close to the natural ostium.	Unremarkable	Normal	Maxillary antrostomy + anterior ethmoidectomy with a sampling of caseous material at maxillary os Clarithromycin for 6 months	Resolution
[24]	33/F	Right nasal obstruction, intermittent epistaxis, purulent rhinorrhea, and headaches for 1 year. CT-round and linear calcification with central lucency resulting in mucosal thickening of the right inferior turbinate and nasal septum with haziness in the right maxillary sinus.	Unremarkable	Normal	ESS Cefdinir 100 mg TID and w mupirocin nasal irrigations	Resolution
[25]	58/F	Fever, left-sided facial pain, swelling, and redness for 3 days. CT- heterogeneous soft tissue density filling left maxilla and ethmoids, with no clear bony erosions.	Diabetes, past episodes of discharging sinuses over the abdominal wall and hip. Tooth extraction and dental implantation 2 years prior to symptoms	Immunocompromised	ESS-a sampling of the maxillary and ethmoid sinus Amoxicillin/clavulanate (2 wks) Endoscopy at follow-up showed granular mucosa with yellow spots in the postoperative cavity. Oral amoxicillin TID (3 months)	Resolution

CT: computed tomography, ESS: endoscopic sinus surgery, IV: intravenous, and TID: three times daily.

## Data Availability

The case report data used to support the findings of this study are included within the article.

## References

[1] Woo H.-J., Chang Hoon B., Song S.-Y., Choi Y. S., Kim Y.-D. (2008). Actinomycosis of the paranasal sinus. *Otolaryngology-Head and Neck Surgery*.

[2] Vorasubin N., Wu A. W., Day C., Suh J. D. (2013). Invasive sinonasal actinomycosis. *The Laryngoscope*.

[3] Per-Lee J. H., Clairmont A. A., Hoffman J. C., McKinney A. S., Schwarzmann S. W. (1974). Actinomycosis masquerading as depression headache: case report--management review of sinus actinomycosis. *The Laryngoscope*.

[4] Sullivan T. J., Aylward G. W., Wright J. E. (1992). Actinomycosis of the orbit. *British Journal of Ophthalmology*.

[5] Wadhera R., Gulati S. P., Garg A., Ghai A., Kumar S. (2008). Frontal sinus actinomycosis presenting as osteomyelitis of frontal bone. *Otolaryngology-Head and Neck Surgery*.

[6] Sama C. B., Mbarga N. F., Oben C. E., Mbarga J. A., Nfor E. K., Angwafo F. F. (15). Massive paediatric cervicofacial actinomycoses masquerading as an ulcerative malignancy. *BMC Infectious Diseases*.

[7] Kolm I., Aceto L., Hombach M., Kamarshev J., Hafner J., Urosevic-Maiwald M. (2014). Cervicofacial actinomycosis: a long forgotten infectious complication of immunosuppression - report of a case and review of the literature. *Dermatology Online Journal*.

[8] Sakuma Y., Yamashita Y., Shiono O., Oridate N. (2016). Actinomycosis arising from the nasal cavity, with rare fatal progression. *BMJ Case Reports*.

[9] Cohn J. E., Lentner M., Li H., Nagorsky M. (2017). Unilateral maxillary sinus actinomycosis with a closed oroantral fistula. *Case Reports in Otolaryngology*.

[10] Fadda G. L., Gisolo M., Crosetti E., Fulcheri A., Succo G. (2014). Intracranial complication of rhinosinusitis from actinomycosis of the paranasal sinuses: a rare case of abducens nerve palsy. *Case Reports in Otolaryngology*.

[11] Saibene A. M., Di Pasquale D., Pipolo C., Felisati G. (2013). Actinomycosis mimicking sinonasal malignant disease. *Case Reports*.

[12] Lerner P. I. (1988). The lumpy jaw. *Infectious Disease Clinics of North America*.

[13] Brook I. (2008). Actinomycosis: diagnosis and management. *Southern Medical Journal*.

[14] Roth M., Montone K. T. (1996). Actinomycosis of the paranasal sinuses: a case report and review. *Otolaryngology-Head and Neck Surgery*.

[15] Bennhoff D. F. (1984). Actinomycosis. *The Laryngoscope*.

[16] Geerlings S. E., Hoepelman A. I. M. (1999). Immune dysfunction in patients with diabetes mellitus (DM). *FEMS Immunology and Medical Microbiology*.

[17] Akhaddar A., Elmostarchid B., Boulahroud O., Elouennass M., Boucetta M. (2009). Actinomycotic brain abscess with osteomyelitis arising from frontal sinusitis. *Internal Medicine*.

[18] Nithyanandam S., D’Souza O., Rao S. S., Battu R. R., George S. (2001). Rhinoorbitocerebral actinomycosis. *Ophthalmic Plastic and Reconstructive Surgery*.

[19] Ohta S., Nishizawa S., Namba H., Sugimura H. (2002). Bilateral cavernous sinus actinomycosis resulting in painful ophthalmoplegia. *Journal of Neurosurgery*.

[20] Damante J., Sant’Ana E., Soares C., Moreira C. (2006). Chronic sinusitis unresponsive to medical therapy: a case of maxillary sinus actinomycosis focusing on computed tomography findings. *Dentomaxillofacial Radiology*.

[21] Cardoso I. C. E., de Mattos Oliveira F., Hochhegger B., Severo L. C. (2015). Sphenoid sinus fungus ball by filaments of actinomycetes and Aspergillus fumigatus. *Mycopathologia*.

[22] Sánchez Legaza E., Cercera Oliver C., Miranda Caravallo J. I. (2013). Actinomicosis de senos paranasales. *Acta Otorrinolaringológica Española*.

[23] Ohbuchi T., Shimajiri S., Kawamura Y., Koga Y., Nakayama T., Suzuki H. (2021). Sinusitis of actinomycosis infection: a case report. *Journal of Medical Investigation*.

[24] Kim S.-D., Kim D.-S., Choi K.-U., Cho K.-S. (2018). Nasal cavity actinomycosis mimicking rhinolith. *Journal of Craniofacial Surgery*.

[25] Varghese L., Cherian L. M., Varghese G. M. (2020). Actinomycosis: an unusual cause of maxillary sinusitis. *Ear, Nose, and Throat Journal*.

